# Left Atrial Strain Outperforms Ventricular Strains in Cardiovascular Outcome Association Across Echocardiography and Magnetic Resonance

**DOI:** 10.1016/j.jacasi.2025.11.013

**Published:** 2026-01-10

**Authors:** Kuang-Chien Chiang, Tetsuji Kitano, Yosuke Nabeshima, Yasufumi Nagata, Seanson C. Ju, Li-Tan Yang, Masaaki Takeuchi

**Affiliations:** a School of Medicine, National Taiwan University, Taipei, Taiwan; b Department of Cardiology, Mie University Hospital, Tsu, Japan; c Department of Cardiovascular Medicine, Saga University, Saga, Japan; d Second Department of Internal Medicine, University of Occupational and Environmental Health, School of Medicine, Kitakyushu, Japan; e Department of Internal Medicine, National Taiwan University Hospital, Taipei, Taiwan; f Department of Laboratory and Transfusion Medicine, Hospital of University of Occupational and Environmental Health, Kitakyushu, Japan

**Keywords:** cardiac magnetic resonance, cohort studies, myocardial strain, risk assessment, speckle tracking echocardiography

## Abstract

**Background:**

Cardiac chamber strain is associated with outcomes in various diseases. However, it remains uncertain whether left ventricular global longitudinal strain (LVGLS), left atrial reservoir strain (LASr), or right ventricular free wall longitudinal strain (RVfwLS) has the most robust association with outcomes.

**Objectives:**

The authors aimed to compare head-to-head the associations of LVGLS, LASr, and RVfwLS with major adverse cardiovascular events (MACE) using 2-dimensional speckle tracking echocardiography (2D-STE) and cardiac magnetic resonance feature tracking (CMR-FT).

**Methods:**

Consecutive patients who underwent clinically indicated echocardiography and cardiac magnetic resonance on the same day were enrolled. Strain was derived from 2D-STE (fully automated and manually edited) and CMR-FT. The primary endpoint was MACE, including cardiac death, heart failure hospitalization, and sustained ventricular tachyarrhythmia.

**Results:**

We included 550 patients (age 65 ± 15 years, 361 [66%] male). Over a median follow-up of 2.2 years (Q1-Q3: 0.9-4.2 years), 78 (14.3%) patients experienced MACE. LVGLS, LASr, and RVfwLS were independently associated with MACE in separate models after adjusted for key clinical variables. Head-to-head comparisons of 3 strain parameters revealed that LASr remained significantly associated with MACE using 2D-STE (both fully automated and manually edited, HR: 0.91; 95% CI: 0.86-0.95) and CMR-FT (HR: 0.91; 95% CI: 0.86-0.96) (all *P* ≤ 0.001). Incremental analysis showed that LASr provided additional value beyond clinical parameters and LVGLS (all *P* < 0.001), while the subsequent addition of RVfwLS did not. Nomogram analyses indicated that LASr had the most significant impact on MACE compared with LVGLS and RVfwLS, as assessed by both 2D-STE and CMR.

**Conclusions:**

Compared with LVGLS and RVfwLS, LASr exhibited the strongest association with MACE, regardless of the imaging modality used. Incorporating LASr as a routine measurement should therefore be considered.

Left ventricular (LV), left atrial (LA), and right ventricular (RV) longitudinal strain have become increasingly accessible in research and clinical settings. Impaired strain values are often associated with poor outcomes.^1^ Some studies have analyzed the prognostic value of LV, LA, and RV longitudinal strain within the same population;^2^, ^3^, ^4^, ^5^, ^6^, ^7^, ^8^, ^9^ however, not all studies conducted head-to-head comparisons of strain in the 3 cardiac chambers, and none have compared LV, LA, and RV strain derived simultaneously from 2-dimensional speckle-tracking echocardiography (2D-STE) and cardiac magnetic resonance (CMR).

Using 2D-STE, left atrial reservoir strain (LASr) showed the strongest associations with mortality compared with left ventricular global longitudinal strain (LVGLS) and right ventricular free wall longitudinal strain (RVfwLS) in cardiac amyloidosis.^4^ However, LVGLS better linked to mortality compared with LASr in pulmonary arterial hypertension.^6^ In patients with heart failure, inconsistent results existed regarding which strain predicted adverse outcomes most efficiently.^2^^,^^3^^,^^5^ Using cardiac magnetic resonance feature tracking (CMR-FT), LA strain showed a stronger association with poor outcomes in dilated cardiomyopathy,^9^ while LVGLS performed better in acute myocarditis.^7^ Due to the discrepant results from different strain modalities across various diseases, the relative strength of outcome associations among LV, LA, and RV longitudinal strain remains unknown.

Accordingly, we measured LVGLS, LASr, and RVfwLS using both 2D-STE and CMR-FT in a large cohort of patients. We aimed to: 1) head-to-head compare LVGLS, LASr, and RVfwLS for their association with major adverse cardiovascular events (MACE); and 2) assess the consistency of these comparisons using 2D-STE and CMR-FT.

## Methods

### Patient population

We conducted a retrospective observational study in consecutive patients who underwent clinically indicated CMR and agreed to undergo transthoracic echocardiography on the same day at the University of Occupational and Environmental Health Hospital, Japan, from January 1, 2014, to December 31, 2022 (Supplemental Figure 1). The elapsed time between 2-dimensional echocardiography and CMR was within 1 hour. Nine patients were excluded due to unavailable data, including missing echocardiographic datasets (n = 7), missing raw data in the TomTec software system (n = 1), and failure to export data into the TomTec Imaging Systems software. During strain measurement, 3 additional patients were excluded due to measurement failure: 2 with extremely poor echocardiographic image quality that precluded strain analysis and 1 with CMR analysis failure due to cycle length error. The institutional ethics committee (Ethics Committee of Medical Research, University of Occupational and Environmental Health, Japan) approved the study protocol, and written informed consent was waived due to the study's retrospective nature (Institutional Review Board number: CR24-059).

### Image acquisition via 2-dimensional transthoracic echocardiography

All study participants underwent standard 2-dimensional transthoracic echocardiography conducted by experienced doctors or sonographers using commercially available ultrasound machines (iE33/EPIQ7G [Philips Healthcare], Vivid7/E95 [GE Healthcare]). Echocardiography was performed in the left lateral decubitus position. We acquired images of apical 4-chamber, 2-chamber, 3-chamber, and RV-focused apical 4-chamber views during 3 consecutive cardiac cycles with breath holding; scan width was adjusted to maximize frame rates.

### Fully automated and manual-edited speckle tracking strain analysis

**Figure 1:**
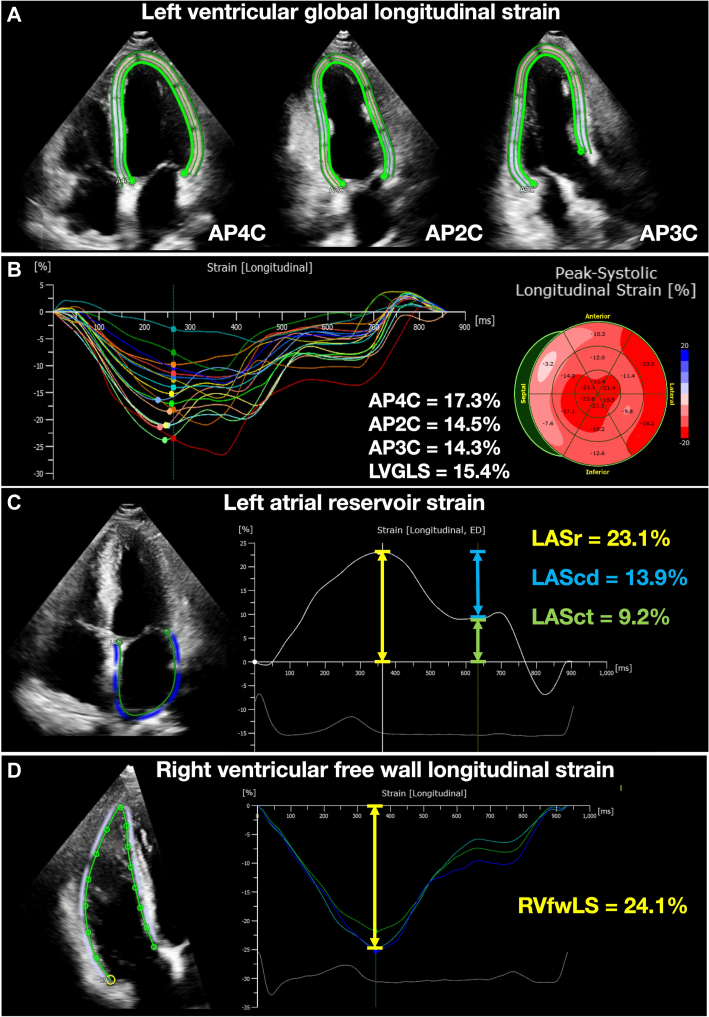
Schema of Fully Automated Speckle Tracking Analysis (A, B) Apical 4-chamber (AP4C), apical 2-chamber (AP2C), and apical 3-chamber (AP3C) views were utilized for assessing left ventricular global longitudinal strain (LVGLS). (C) The AP4C containing the whole part of left ventricle and left atrium was used to measure left atrial reservoir strain (LASr), and (D) a right ventricular-focused AP4C view was employed for right ventricular free wall longitudinal strain (RVfwLS). The software automatically generated strain curves of the 3 cardiac chambers. ED = end-diastole; LAScd = left atrial conduit strain; LASct = left atrial contractile strain.

We conducted strain analyses using fully automated strain software (AutoSTRAIN; TomTec Imaging Systems) (Figure 1). For LVGLS, we selected apical 4-chamber, 2-chamber, and 3-chamber views. The software utilized a knowledge-based artificial intelligence algorithm to automatically register each apical view, detect the second cardiac cycle, determine the LV endocardial border, and perform speckle tracking analyses. We retrieved the results as the fully automated speckle tracking strain values. For LASr and RVfwLS, we conducted similar analyses: for LASr, apical 4-chamber which encompassed whole part of both LV and LA was used, and for RVfwLS, the RV-focused apical 4-chamber view was used for analysis. Image quality was evaluated based on the number of visible segments (Supplemental Methods).

We also performed manually edited speckle tracking analysis in each patient (Supplemental Figure 2). Specifically, we manually selected the LV end-diastolic and end-systolic phases.^10^ Then, we edited the endocardial borders of the LV and RV at the LV end-systole, and that of the LA at the LV end-diastole. The software then performed new speckle tracking analyses during the second cardiac cycle. Manual correction of endocardial border was performed when required. We retrieved the calculated value as the manually edited strain.

### CMR imaging acquisition and feature tracking strain analysis

**Figure 2:**
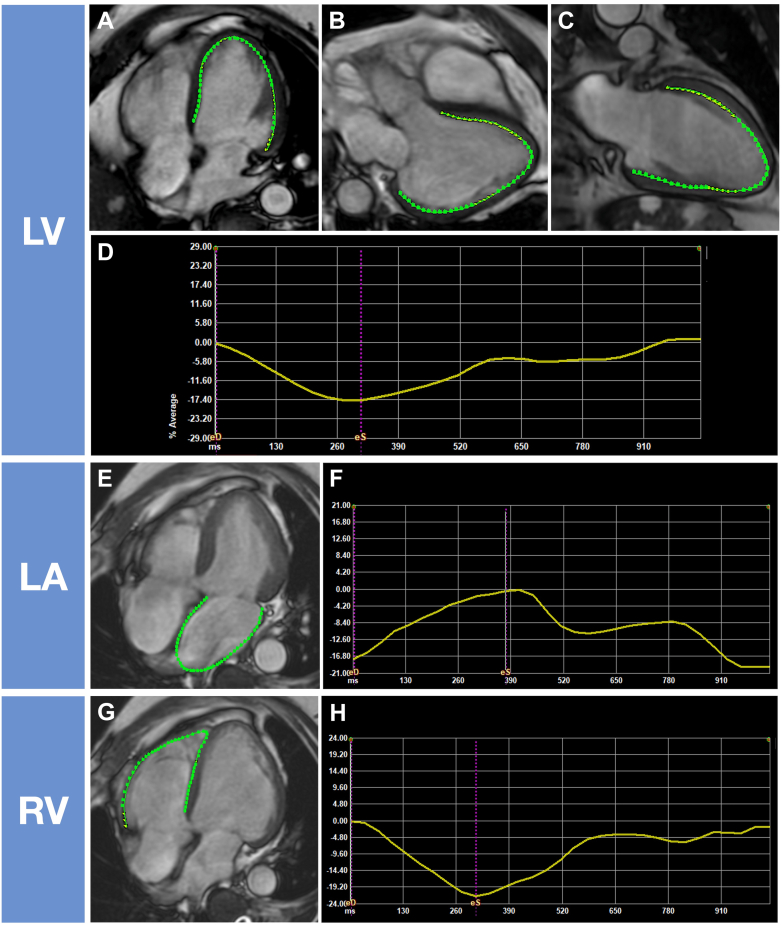
Schema of Cardiac Magnetic Resonance Feature Tracking Analysis The endocardial borders of (A-C) the left ventricle (LV), (E) the left atrium (LA), and (G) the right ventricle (RV) were automatically detected in apical views and manually adjusted when needed. (D, F, H) Strain curves were constructed automatically.

CMR was performed by a 3.0T scanner (Discovery 750W or SIGNA premier; GE Healthcare) with a phase-array cardiovascular coil. We identified the long-axis of the heart using retrospective, electrocardiographically gated and localizing spin‒echo sequences. Steady-state free precession dynamic gradient-echo cine loops were acquired using parallel imaging techniques during 10- to 15-second breath holding, with the following parameters: slice thickness of imaging planes of 8 mm, field of view of 40 × 40 cm, scan matrix of 200 × 160, flip angle of 50°, repetition time of 2.8 ms, echo time of 1.7 ms, and number of reconstructed cardiac phases of 20 to 30. Steady-state free precession CMR images were obtained from stacked LV short-axis views, as well as the 3 apical long-axis views (apical 4-chamber, 2-chamber, and 3-chamber). We obtained LVGLS, LASr, and RVfwLS using a CMR-FT software (2DCPA MR; TomTec Imaging Systems GmbH) (Figure 2). For LVGLS, we utilized the 3 apical long-axis cine steady-state free precession images; the LV endocardial border at LV end-diastole was obtained after clicking 3 anatomical landmarks (both sides of mitral annulus and LV apex). Manual adjustments were performed when needed. Subsequently, the software performed feature tracking analysis. We then obtained LVGLS by averaging the strain values in each image. Similar analyses were conducted for feature tracking values of LASr and RVfwLS under the same apical 4-chamber view.

### Clinical data and follow-up outcomes

We retrieved patient age, sex, anthropometric data, the NYHA functional class, and comorbid conditions from electronic medical records. Charlson Comorbidity Index (CCI) was calculated.^11^

Follow-up information was obtained by electronic medical records or telephone interview when necessary. The primary endpoint was MACE, including cardiac death, heart failure hospitalization, nonfatal myocardial infarction, and sustained ventricular tachycardia or ventricular fibrillation. If more than 1 event occurred in the same patient, the earliest event was used for the analysis. The follow-up duration was calculated from the date of 2D TTE/CMR to the event or censored date. The end of follow-up was December 2023.

### Statistical analysis

Variables were expressed as mean ± SD, median (Q1-Q3), or n (%). The intra- and interclass correlation coefficient was used to represent reproducibility of strain values in 25 randomly selected patients. Histograms were utilized to visualize the distribution of strain values obtained from different methodologies. Pearson correlation was performed to assess the correlation of myocardial strain between 2D-STE and CMR-FT. Bias and limit of agreement were evaluated using Bland-Altman analysis.

Predefined strain cutoffs derived from previous studies (Supplemental Table 1)^12^, ^13^, ^14^, ^15^, ^16^, ^17^ were used to stratify patients into 2 groups (higher and lower strain groups) for Kaplan-Meier survival analysis, with differences compared using the log-rank test. Adjusted Kaplan-Meier curves were calculated using direct standardization. Specifically, multivariable Cox models were constructed including age, sex, NYHA functional class, CCI, and strain group as covariates. The counterfactual conditional survival for each individual was then estimated under the hypothetical scenarios that all patients belonged to either the higher- or lower-strain group. The population-averaged survival probabilities were subsequently obtained as the arithmetic mean of these individual estimates and was compared using the Pepe and Fleming test.

Both univariable and multivariable Cox proportional hazards models were employed to calculate HRs and 95% CIs for established factors (age, sex, NYHA functional class, CCI) and all strain parameters. To keep the number of covariates within event number divided by 10, we categorized NYHA functional class into 2 groups (NYHA functional class I or II and III or IV).^18^ LVGLS, LASr, and RVfwLS, obtained using the same methodology, were incorporated simultaneously into multivariable Cox models to head-to-head compare their associations with MACE. The proportional hazards assumption was assessed using Schoenfeld residual–based statistics. Stratification by sex was further applied due to violation of the proportional hazards assumption (Supplemental Table 2).

Nested Cox regression models were employed to evaluate the incremental values of LVGLS, LASr, and RVfwLS. Initially, a baseline model including age, sex, NYHA functional class, and CCI was constructed. We performed a stepwise inclusion of LVGLS, LASr, and RVfwLS to evaluate their impact on the predicting ability of the model. Subsequent models incorporating alternative sequences of echocardiographic strain were also constructed. All incremental Cox models were stratified by sex to account for violation of the proportional hazards assumption (Supplemental Table 2). Differences in likelihood ratio statistics for the nested models were analyzed, and changes in chi-square statistics were calculated to determine the incremental prognostic value of the strain parameters. The C-index was utilized to assess model performance and discrimination.

Predicted risks at 2-year follow-up were derived from the nested Cox models to calculate continuous net reclassification improvement and integrated discrimination improvement. Specifically, net reclassification improvement measures the increased event rate among patients reclassified as higher risk and the decreased event rate among those reclassified as lower risk, while integrated discrimination improvement compares the average difference in predicted risk for patients who developed MACE and those who did not. Positive and statistically significant net reclassification improvement and integrated discrimination improvement values indicate a net benefit of incorporating myocardial strain into the nested Cox models. Furthermore, decision curve analysis was performed to evaluate the net clinical benefit of each model across a range of risk thresholds for clinical intervention.

To better visualize and compare the association of each strain with MACE, we constructed nomograms based on key variables in the previously mentioned multivariable Cox regression analysis for the predictions of 1-, 3-, and 5-year event-free survival, with variables rescaled to a 100-point range. The C-index with correction of resampling by the bootstrapping method was calculated to verify the reliability of nomogram. All statistical analyses were conducted using R software (version 4.0.2; R Foundation for Statistical Computing). A *P* value <0.05 was considered statistically significant.

## Results

### Patient characteristics and strain analysis

Our final cohort included 550 patients, who were predominantly male, with a mean age of 65 years (Table 1). Upon CMR/TTE examination, 57 (10%) patients were NYHA functional class III or IV. The most prevalent indications for CMR were ischemic heart disease (33% [n = 183 of 550]) and secondary cardiomyopathy (30% [n = 163 of 550]), followed by dilated cardiomyopathy (9% [n = 49 of 550]) and valvular heart diseases (9% [n = 48 of 550]).

**Table 1:** Baseline Clinical, Echocardiographic, and Myocardial Strain Characteristics in the Study Population (N = 550)

Age, y	65 ± 15
Male	361 (66)
Body surface area, m^2^	1.63 ± 0.20
NYHA functional class	
I	245 (45)
II	247 (45)
III/IV	57 (10)
Smoking status	
Quitted	180 (35)
Current smoker	97 (19)
Comorbidities	
Coronary artery disease	208 (40)
Chronic kidney disease	213 (41)
Diabetes mellitus	146 (28)
Hypertension	283 (54)
Hyperlipidemia	216 (42)
Atrial fibrillation	60 (11)
CCI	4 (3-6)
Clinical settings	
Inpatient	329 (60)
Outpatient	221 (40)
Vital signs and laboratory data	
Heart rate, beats/min	68 ± 13
Systolic blood pressure, mm Hg	129 ± 23
Diastolic blood pressure, mm Hg	73 ± 13
Hemoglobin (n = 543), g/dL	12.93 ± 5.43
Creatinine (n = 544)	0.91 (0.75-1.14)
Estimated glomerular filtration rate (n = 538), mL/min	59 ± 22
Low-density lipoprotein (n = 492), mg/dL	93 ± 33
Blood glucose (n = 512), mg/dL	128 ± 55
B-type natriuretic peptide (n = 321), pg/mL	205 (67-471)
N-terminal pro–B-type natriuretic peptide (n = 346), pg/mL	1086 (277-3212)
Indications for CMR examinations	
Ischemic heart disease	183 (33)
Secondary cardiomyopathy	163 (30)
Dilated cardiomyopathy	49 (9)
Valvular heart disease	48 (9)
≥moderate AR^a^	13 (2)
≥moderate AS	16 (3)
≥moderate MR^a^	5 (1)
Hypertrophic cardiomyopathy	32 (6)
Pulmonary hypertension	20 (4)
Myocarditis	7 (1)
Others	48 (8)
Echocardiographic parameters	
LV end-diastolic volume index, mL/m^2^	73 ± 26
LV end-systolic volume index, mL/m^2^	40 ± 25
LV ejection fraction, %	48 ± 17
LA volume index, mL/m^2^	48 ± 34
E, cm/s	70 ± 30
A, cm/s	70 ± 30
E/A	0.87 (0.65-1.25)
Average e′, cm/s	6.3 ± 2.3
E/e′	12.4 ± 6.2
TR pressure gradient, mm Hg	26.3 ± 10.8
Right atrial pressure, mm Hg	6.7 ± 2.8
TAPSE, mm	16.8 ± 5.3
FAC, %	43.2 ± 16.8
Myocardial strain parameters, %	
LVGLS_auto_	13.6 ± 4.8
LVGLS_edit_	13.1 ± 4.8
LVGLS_ft_	11.2 ± 4.6
LASr_auto_	23.6 ± 12.9
LASr_edit_	20.5 ± 10.5
LASr_ft_	13.9 ± 7.4
RVfwLS_auto_	19.8 ± 6.8
RVfwLS_edit_	19.2 ± 6.3
RVfwLS_ft_	19.8 ± 7.6
Cardiac magnetic resonance parameters	
LV end-diastolic volume index, mL/m^2^	102 ± 42
LV end-systolic volume, mL/m^2^	66 ± 42
LV ejection fraction, %	39 ± 15
LV mass index, g/m^2^	74 ± 25
RV end-diastolic volume index, mL/m^2^	116.2 ± 39.7
RV end-systolic volume index, mL/m^2^	40 ± 19
RV ejection fraction	45 ± 11
Myocardial scar (n = 158), %	12 (7-22)
Extracellular volume (n = 90 of 143), %	37 ± 15

Values are mean ± SD, n (%), or median (Q1-Q3). Secondary cardiomyopathy included cardiac sarcoidosis, cardiac amyloidosis, hypertensive cardiomyopathy, ampulla cardiomyopathy, chemotherapy related cardiac dysfunction, tachycardia cardiomyopathy, and alcoholic cardiomyopathy. “Auto” indicates “fully automated,” “edit” indicates “manually edited,” and “ft” indicates “feature tracking.”

A = atrial diastolic transmitral flow velocity; AR = aortic regurgitation; AS = aortic stenosis; CCI = Charlson Comorbidity Index; CMR = cardiac magnetic resonance; E = early diastolic transmitral flow velocity; E/A = ratio of early to atrial diastolic transmitral flow velocity; E/e′ = ratio of early diastolic transmitral flow velocity to mitral annulus early diastolic velocity; e′ = mitral annulus early diastolic velocity; FAC = fractional area change; LA = left atrial; LASr = left atrial reservoir strain; LV = left ventricular; LVGLS = left ventricular global longitudinal strain; MR = mitral regurgitation; RVfwLS = right ventricular free wall longitudinal strain; TAPSE = tricuspid annular plane systolic excursion; TR = tricuspid regurgitation.

a One patient had ≥moderate AR and ≥moderate MR simultaneously.

In terms of fully automated speckle tracking echocardiography analysis, we successfully performed LVGLS, LASr, and RVfwLS analysis in 97% (n = 532 of 550), 96% (n = 527 of 550), and 88% (n = 483 of 550) of patients, respectively. Feasibility improved to 98% (n = 538 of 550), 99% (n = 542 of 550), and 91% (n = 500 of 550) for manually edited analysis, respectively. In CMR-FT, the corresponding feasibility was 94% (n = 516 of 550), 94% (n = 519 of 550), and 94% (n = 516 of 550), respectively. Supplemental Figure 3 displays histograms of strain in each chamber analyzed using different modalities. Compared with LVGLS and RVfwLS, LASr showed a wider distribution, particularly with 2D-STE, and this was especially prominent with fully automated LASr.

In Supplemental Table 3, 2D-STE and CMR-FT demonstrated strong correlations for LVGLS (r = 0.79 [95% CI: 0.76-0.82] for automated and r = 0.81 [95% CI: 0.77-0.84] for manually edited strain) and moderate correlations for LASr (r = 0.67 [95% CI: 0.62-0.72] for automated and r = 0.67 [95% CI: 0.62-0.71] for manually edited strain) and RVfwLS (r = 0.48 [95% CI: 0.41-0.55] for automated strain and r = 0.52 [95% CI: 0.45-0.58] for manually edited strain). Bland-Altman analysis revealed wide limits of agreement across all strain parameters, largest for LASr (mean bias: 9.7 [LOA:−9.2 to 28.6] for automated and 6.7 [LOA: −8.7 to 22.0] for manually edited strain), followed by RVfwLS (−0.1 [LOA: −14.6 to 14.4] for automated and −0.6 [LOA: −14.3 to 13.1] for manually edited strain), and smallest for LVGLS (2.4 [LOA: −3.5 to 8.3] for automated and 1.9 [LOA: −3.7 to 7.6] for manually edited strain). The wide LOA indicate that 2D-STE and CMR-FT were not interchangeable. The inter- and intraobserver variability are presented in Supplemental Table 4.

### Associations between individual chamber strain and MACE

During a median follow-up of 2.2 years (Q1-Q3: 0.9-4.2 years), 14.3% (n = 78 of 550) patients reached the primary endpoint (cardiac death [n = 19], heart failure hospitalization [n = 51], and sustained ventricular tachycardia/ventricular fibrillation [n = 8]). Univariable analysis showed that clinical parameters, including age, body surface area, NYHA functional class, CCI, and systolic and diastolic blood pressures, were associated with MACE (Supplemental Table 5). Regardless of the methodology used, LVGLS, LASr, and RVfwLS remained significantly associated with MACE after adjustment for age, sex, NYHA functional class, and CCI. The models were stratified by sex due to violation of the proportional hazards assumption (Supplemental Table 2).

**Figure 3:**
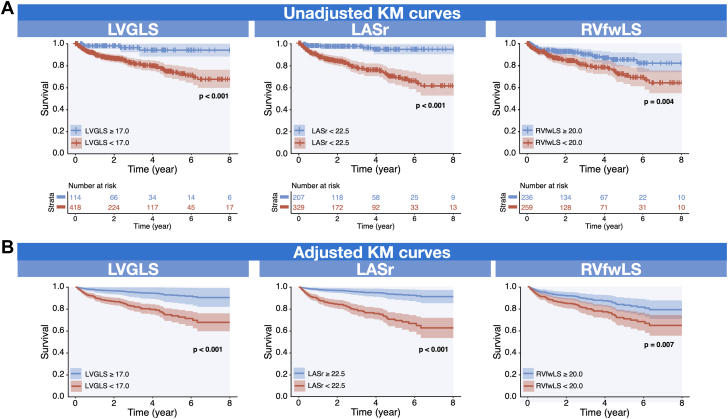
Kaplan-Meier Analyses of Multichamber Strain Using Manually Edited Speckle Tracking Echocardiography Patients with lower LVGLS, LASr, and RVfwLS were associated with higher rates of major adverse cardiovascular events (A) before and (B) after adjustment. Adjustments were made for age, sex, NYHA functional class, and Charlson Comorbidity Index. KM = Kaplan-Meier; other abbreviations as in Figure 1.

Figure 3 and Supplemental Figure 4 demonstrate Kaplan-Meier survival curves using 2D-STE strain for patient stratification. The cutoff values were 17.0% for LVGLS,^14^ 22.5% for LASr,^17^ and 20.0% for RVfwLS,^15^ respectively. Patients with lower LVGLS, LASr, or RVfwLS were associated with higher rates of MACE, using either fully automated (Supplemental Figure 4A) or manually edited (Figure 3A) strain (all log-rank *P <* 0.005). After adjustment for age, sex, NYHA functional class, and CCI, patients with lower LVGLS, LASr, or RVfwLS still experienced a significantly higher incidence of MACE (all *P <* 0.001) (Figure 3B, Supplemental Figure 4B).

For CMR-FT strain (Supplemental Figure 5), we utilized cutoff values of 12.5% for LVGLS,^16^ 14.8% for LASr,^13^ and 17.2% for RVfwLS,^12^ respectively. Similarly, we observed a higher rate of MACE in patients with lower CMR-FT strain values (*P* < 0.001). After adjusting for age, sex, NYHA functional class, and CCI, patients with lower strain values remained at higher risk for MACE (all *P <* 0.001).

### Head-to-head comparison of multichamber strain

Multivariable analysis, including previously mentioned covariates, LVGLS, LASr, and RVfwLS, revealed that only LASr, and not LVGLS or RVfwLS, was independently associated with MACE in fully automated and manually edited 2D-STE, as well as in CMR-FT (Table 2). Stratification by sex was applied to account for violation of the proportional hazards assumption (Supplemental Table 2). Across all strain modalities, the variance inflation factors were below 2.5, indicating no collinearity.

**Table 2:** Multivariable Cox Proportional Hazards Analysis for the Association With Major Adverse Cardiovascular Events

	Fully Automated Analysis Model			Manually Edited Analysis Model			CMR Feature Tracking Analysis Model		
	HR	95% CI	*P* Value	HR	95% CI	*P* Value	HR	95% CI	*P* Value
Age	1.02	0.99-1.05	0.200	1.02	0.99-1.05	0.187	1.02	0.99-1.05	0.165
NYHA functional class III or IV	2.36	1.28-4.37	0.006	2.15	1.18-3.94	0.013	2.42	1.34-4.37	0.003
CCI	1.07	0.94-1.22	0.283	1.09	0.97-1.23	0.160	1.06	0.93-1.21	0.374
LVGLS_auto_	1.01	0.93-1.10	0.741						
LASr_auto_	0.91	0.87-0.95	<0.001						
RVfwLS_auto_	1.00	0.95-1.05	0.978						
LVGLS_edit_				1.00	0.93-1.09	0.950			
LASr_edit_				0.91	0.86-0.95	<0.001			
RVfwLS_edit_				0.98	0.93-1.03	0.388			
LVGLS_ft_							0.95	0.88-1.02	0.132
LASr_ft_							0.91	0.86-0.96	<0.001
RVfwLS_ft_							0.98	0.95-1.02	0.371

All models were stratified by sex. “Auto” indicates “fully automated,” “edit” indicates “manually edited,” and “ft” indicates “feature tracking.”

Abbreviations as in Table 1.

**Figure 4:**
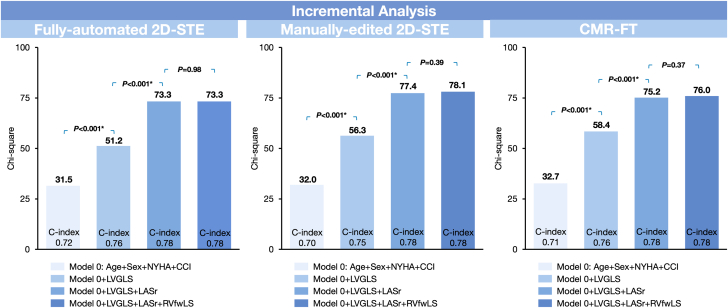
Incremental Value of Multichamber Strain Across Different Imaging Modalities Model 0 included age, NYHA functional class, Charlson comorbidity index (CCI), and stratification by sex. Across all imaging modalities, the stepwise addition of LASr to the LVGLS-based model showed added value for major adverse cardiovascular events. In contrast, RVfwLS did not provide additional value when added to the LVGLS and LASr-combined model (model 0 +LVGLS +LASr). CMR-FT = cardiac magnetic resonance feature tracking; 2D-STE = 2-dimensional speckle tracking echocardiography; other abbreviations as in Figure 1.

Table 3 presents the nested Cox regression analyses using 3 methodologies for strain assessment, with all Cox models stratified by sex due to violation of the proportional hazards assumption (Supplemental Table 2). Model 0 comprises conventional clinical predictors, including age, sex, NYHA functional class, and CCI. LVGLS exhibited incremental value for MACE on top of model 0 (Figure 4). The stepwise addition of LASr to the LVGLS-based model showed added value for MACE, regardless of the strain assessment modality (Figure 4). In contrast, adding RVfwLS to the LVGLS-based model exhibited significant incremental value only with CMR-FT (Table 3). Nevertheless, RVfwLS failed to provide additional value when added to the LVGLS and LASr-combined model (model 0 +LVGLS +LASr), regardless of the modality used.

**Table 3:** Nested Cox Regression Models Assessing Incremental Value of Myocardial Strain Across Imaging Modalities

	LR Statistic	AIC	C-Statistic (95% CI)	*P* Value
Fully automated speckle tracking echocardiography				
Model 0: age + NYHA + CCI	31.5	608	0.72 (0.65-0.78)	—
Model 1: model 0 +LVGLS	51.2	590	0.76 (0.69-0.82)	<0.001^a^
Model 2: model 0 +LASr	73.1	568	0.78 (0.72-0.85)	<0.001^a^
Model 3-1: model 1 + LASr	73.3	570	0.78 (0.72-0.85)	<0.001^b^
Model 3-2: model 2 + LVGLS				0.717^c^
Model 4: model 1 +RVfwLS	51.9	591	0.76 (0.69-0.82)	0.404^b^
Model 5-1: model 3-1+ RVfwLS	73.3	572	0.78 (0.72-0.85)	0.978^d^
Model 5-2: model 4 + LASr				<0.001^e^
Manually edited speckle tracking echocardiography				
Model 0: age + NYHA + CCI	32.0	677	0.70 (0.64-0.77)	—
Model 1: model 0 +LVGLS	56.3	655	0.75 (0.70-0.81)	<0.001^a^
Model 2: model 0 +LASr	77.3	634	0.78 (0.73-0.84)	<0.001^a^
Model 3-1: model 1 + LASr	77.4	636	0.78 (0.73-0.84)	<0.001^b^
Model 3-2: model 2 + LVGLS				0.814^c^
Model 4: model 1 +RVfwLS	59.6	653	0.76 (0.70-0.82)	0.069^b^
Model 5-1: model 3-1+ RVfwLS	78.1	637	0.78 (0.72-0.84)	0.386^d^
Model 5-2: Model 4 + LASr				<0.001^e^
CMR feature tracking				
Model 0: age + NYHA + CCI	32.7	682	0.71 (0.65-0.78)	—
Model 1: model 0 +LVGLS	58.4	658	0.76 (0.70-0.82)	<0.001^a^
Model 2: model 0 +LASr	72.3	644	0.77 (0.71-0.83)	<0.001^a^
Model 3-1: model 1 + LASr	75.2	643	0.78 (0.72-0.84)	<0.001^b^
Model 3-2: model 2 + LVGLS				0.086^c^
Model 4: model 1 +RVfwLS	62.9	655	0.77 (0.71-0.82)	0.035^b^
Model 5-1: model 3-1+ RVfwLS	76.0	644	0.78 (0.73-0.84)	0.367^d^
Model 5-2: model 4 + LASr				<0.001^e^

All Cox models are stratified by sex. *P* values for the comparison of LR statistics between models are represented using different superscripts.

AIC = Akaike information criterion; LR = likelihood ratio; other abbreviations as in Table 1.

a LR test with model 0.

b LR test with model 1.

c LR test with model 2.

d LR test with model 3.

e LR test with model 4.

Supplemental Figure 6 displays an alternative model sequence where LASr was added first to model 0. Neither further addition of LVGLS nor RVfwLS by any modality showed additional value over model 0 +LASr.

Supplemental Table 6 illustrates the integrated discrimination improvement and net reclassification improvement values of the nested Cox models. Across all models, the inclusion of LASr consistently demonstrated significant improvement in model performance, as reflected by the positive integrated discrimination improvement and net reclassification improvement values with 95% CIs above zero. In contrast, although LVGLS contributed to significant integrated discrimination improvement and net reclassification improvement when added to model 0, it did not provide additional improvement when LASr was already included. Similarly, RVfwLS did not further enhance model performance beyond that provided by LVGLS or LASr.

Supplemental Figure 7 displays the decision curve analyses for the nested Cox models across imaging modalities. Across all modalities, the inclusion of any myocardial strain parameter yielded greater net clinical benefit compared with the baseline model. Notably, incorporation of LASr, either alone or in combination with other strain parameters, consistently provided additional net clinical benefit across a wide range of probability thresholds.

### Nomograms and event-free survival of MACE

**Figure 5:**
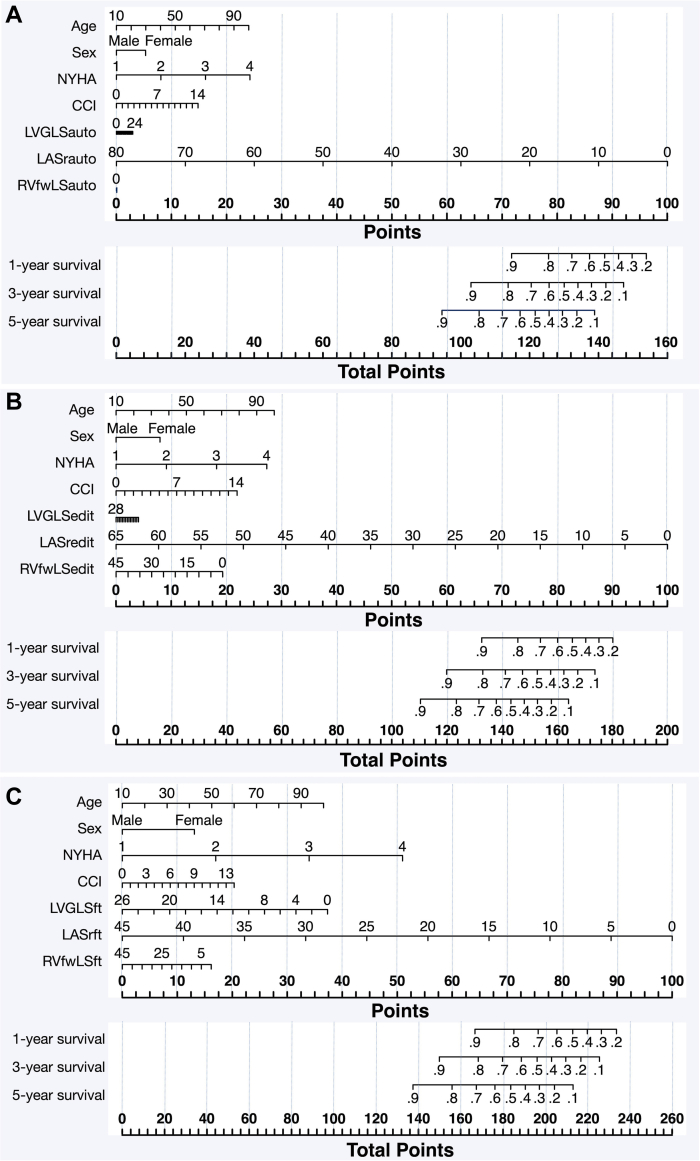
Nomogram for the Occurrence of Major Adverse Cardiovascular Events Nomogram for the 1-, 3-, and 5-year occurrence of major adverse cardiovascular events. LASr assessed by (A) fully automated and (B) manually edited 2D-STE, and (C) CMR-FT consistently exhibited the greatest impact on prognosis. “Auto” indicates “fully automated 2D-STE,” “edit” indicates “manually edited 2D-STE,” and “ft” indicates “CMR-FT.” Abbreviations as in Figure 1.

Figure 5 demonstrates the nomograms constructed by key variables from the multivariable Cox proportional hazards models. Among all fully automated 2D-STE strain, 1-unit decrease in LASr had the most impact on the total score in the nomogram (Figure 5A). Notably, the contribution of both LVGLS and RVfwLS seemed negligible. The bootstrap-corrected C-index for this nomogram was 0.79 (95% CI: 0.74-0.85), indicating acceptable model performance.

When using manually edited 2D-STE strain for nomogram construction, LASr remained the most robust determinant for MACE (Figure 5B), followed by RVfwLS, with LVGLS contributing the least. The C-index was 0.79 (95% CI: 0.73-0.85). In the nomogram constructed using CMR-FT strain, LASr remained to be the most robust determinant for MACE (Figure 5C); the effect of LVGLS became more prominent, exceeding that of RVfwLS. The C-index was 0.79 (95% CI: 0.73-0.84), suggesting fair model performance.

## Discussion

This is the first study to head-to-head compare multichamber myocardial strain assessed by 2D-STE and CMR-FT for their associations with MACE in a large cohort of patients (Central Illustration). The principal findings were the following. First, patients with lower LVGLS, LASr, and RVfwLS, whether derived from 2D-STE and CMR-FT, consistently exhibited higher risks of MACE, as shown by Kaplan-Meier survival analysis. Second, direct comparisons of LVGLS, LASr, and RVfwLS showed that LASr remained a robust determinant for MACE regardless of the imaging modalities used. Third, LASr provided significant incremental value for predicting MACE when added to models that included clinical factors and LVGLS. However, the further addition of RVfwLS did not enhance the predictive power of the models. Fourth, in integrated discrimination improvement and net reclassification improvement analyses, LASr consistently improved model performance beyond LVGLS and RVfwLS. Fifth, decision curve analyses indicated the greatest net clinical benefit when LASr was incorporated into the models. Sixth, nomogram analyses demonstrated that LASr was the most influential indicator for calculating MACE risk, outperforming LVGLS and RVfwLS. These findings were consistent across 2D-STE and CMR-FT modalities.

**Central Illustration:**
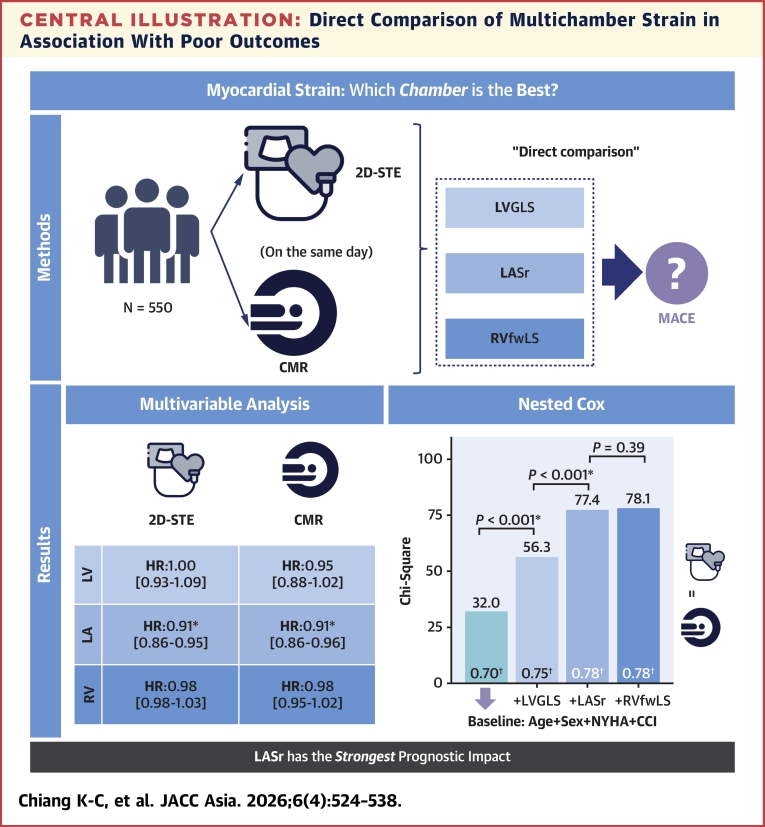
Direct Comparison of Multichamber Strain in Association With Poor Outcomes In multivariable Cox analyses, direct comparison of multichamber strain suggested that left atrial reservoir strain (LASr) remained a robust determinant of major adverse cardiovascular events (MACE). Nested Cox models demonstrated that LASr provided significant incremental prognostic value when added to the model including clinical factors and left ventricular global longitudinal strain (LVGLS), whereas further addition of right ventricular free wall longitudinal strain (RVfwLS) did not yield additional benefit. ∗A *P* value <0.05 was considered statistically significant. †C-index of the nested Cox regression models. CCI = Charlson Comorbidity Index; CMR = cardiac magnetic resonance; LA = left atrium; LV = left ventricle; RV = right ventricle; 2D-STE = 2-dimensional speckle tracking echocardiography.

### Previous study

Few studies have directly compared the prognostic strength of LV, LA, and RV strain, and have reported conflicting results. In patients with dilated cardiomyopathy, Xiang et al^9^ suggested that LA conduit strain has a stronger prognostic ability than LVGLS and RV global longitudinal strain when assessed using CMR. Another study suggested that LASr exhibited the strongest association with survival compared with LVGLS or RVfwLS in cardiac amyloidosis.^4^ In patients with heart failure with preserved ejection fraction, Freed et al^2^ found that LASr outperformed LVGLS and RVfwLS in predicting cardiovascular death and hospitalization. Nevertheless, Rothschild et al^3^ challenged these findings, demonstrating the incremental value of RV global longitudinal strain to LVGLS and LASr for predicting all-cause death in heart failure with preserved ejection fraction patients. Moreover, LASr also failed to outperform other LVGLS or RV global longitudinal strain in patients with pulmonary arterial hypertension^6^ and acute myocarditis.^7^

### Current study

To our knowledge, this is the first research to conduct a head-to-head comparison of LVGLS, LASr, and RVfwLS and their associations with MACE using both 2D-STE and CMR-FT within the same cohort of patients. Using multiple statistical approaches, we demonstrated that LASr consistently outperformed LVGLS and RVfwLS in its association with MACE, regardless of the imaging modalities employed.

LASr reflects structural and pathophysiological alterations.^19^ First, LASr represents LA compliance and relaxation capability as LA receives blood from pulmonary veins during LV systole. Additionally, by measuring peak oxygen consumption, Freed et al^2^ demonstrated the important role of LASr in augmenting cardiac output during exercise in patients with heart failure with preserved ejection fraction. Studies utilizing both 2D-STE^20^ and CMR^21^ supported using impaired LASr as a surrogate marker of biopsy-proven LA myocardial fibrosis, a precursor of cardiovascular diseases and adverse outcomes.^22^ Our study, which included patients with significant LA remodeling signified by enlarged LA volume index and impaired LASr, resonates with previous observations demonstrating the independent associations of LASr with MACE (Figure 3, Supplemental Figures 4 and 5).

The LV^19^ and RV^23^ also interact interdependently with LA. As the LV contracts, the descending cardiac base causes passive LA expansion, generating negative pressure that draws blood from pulmonary veins into the LA.^19^ When LV diastolic dysfunction occurs, LASr serves as a sensitive marker, reflecting the severity of diastolic dysfunction by capturing reduced LA compliance.^24^ On the other hand, RV dysfunction can result in insufficient preload of the left heart due to decreased RV output and impaired LV filling.^23^ Rothschild et al^3^ and Kishiki et al^6^ supported these mechanisms, suggesting that RV global longitudinal strain and LVGLS have more robust associations with outcomes compared with LASr. However, the RV global longitudinal strain model was adjusted for LVGLS and LASr by Rothschild et al, leaving it uncertain whether LVGLS and LASr are independently associated with outcomes. Unlike our cohort with only 4% (n = 20 of 550) patients having pulmonary arterial hypertension, Kishiki et al exclusively studied patients with severe pulmonary arterial hypertension and impaired RV function (RV fractional area change, 32%), which may explain why RVfwLS remained significantly associated with mortality.

In our study, LASr was the sole determinant of MACE in the head-to-head comparison of 3 strain parameters regardless of imaging modalities used. Our analysis showed no collinearity among LVGLS, RVfwLS, and LASr, indicating that LASr was the most robust marker and provided incremental prognostic value beyond what is offered by LVGLS and RVfwLS. Interestingly, Xiang et al^9^ also found that CMR-FT derived LA strain outperformed LVGLS and RVfwLS, which aligns with our findings. Although our results differed from the study by Vos et al,^7^ who found LVGLS to be better correlated with MACE compared with LASr in acute myocarditis, this may be because myocarditis mainly affects ventricles rather than atria during the acute phase.

In summary, the LA plays a crucial role in modulating LV filling and optimizing cardiac output.^19^ Impaired LASr, a critical surrogate marker of LA function and early diastolic dysfunction, is associated with MACE in various cardiovascular diseases,^24^ as demonstrated in our study, which included a heterogeneous patient population.

### Clinical implications

Our study may have several clinical implications. First, LASr values below the lower limit for healthy individuals should raise concern for clinicians to closely monitor the patient or initiate timely treatment. Second, as automated strain software becomes more widely available, LASr measurements could be performed more efficiently and incorporated into routine echocardiography exams. Last, LASr could potentially be incorporated as a prognostic indicator in future clinical guidelines.

### Study limitations

First, there was inherent selection bias related to the retrospective study design and our patients’ availability to undergo CMR. Second, the single-center nature of our study may limit generalizability, as the prevalence and spectrum of underlying cardiovascular diseases in our cohort may not reflect those in the general population. Although we included consecutive patients regardless of underlying pathology to reflect real-world clinical practice, this approach limits our ability to account for the potential effects of disease-specific treatments on strain parameters. Larger-scale studies that enable subgroup analyses or disease-specific investigations will be needed to address this limitation. Third, large cohort studies establishing cutoff values for abnormal CMR-FT strain are still lacking. Fourth, we assessed LASr only in the apical 4-chamber view instead of using biplane strain values. However, we deemed this approach acceptable as current standardizing LA strain imaging guidelines support it.^25^ Finally, our study did not have nonforeshortened LA views for 2D-STE^26^ and CMR-FT^27^ analysis. Future investigations will be necessary to determine whether similar conclusions apply when employing 3-dimensional echocardiography analyses.

## Conclusions

In this diverse patient cohort, LASr demonstrated the strongest association with MACE, offering significant incremental value over LVGLS and RVfwLS. Furthermore, the findings were consistent across both 2D-STE and CMR-FT analyses, reinforcing LASr as a robust indicator for outcome association. Incorporating LASr into clinical practice for identifying high-risk patients should be considered, as it may enhance early detection and provide tailored management strategies.

## Funding Support and Author Disclosures

This research was supported by the National Science and Technology Council (NSTC 114-2314-B-002-220), Taipei, Taiwan and National Taiwan University Hospital (114-IF0006), Taipei, Taiwan. The authors have reported that they have no relationships relevant to the contents of this paper to disclose.
