# Supplementary material for: Left Atrial Strain Outperforms Ventricular Strains in Cardiovascular Outcome Association Across Echocardiography and Magnetic Resonance
Source: JACC Asia. 2026 Jan 10;6(4):524–38. doi: 10.1016/j.jacasi.2025.11.013 (PMC13080744; doi:10.1016/j.jacasi.2025.11.013)
Supplement: Supplemental Material [file mmc1.docx]

**Supplemental Materials**

|  | **Page** |
| --- | --- |
| **Supplemental Methods.** Definitions of image quality in two-dimensional echocardiography | 2 |
| **Supplemental Table 1.** Cutoff values of myocardial strain for patient stratification according to previous publications | 3 |
| **Supplemental Table 2.** Validation of the proportional hazard assumption using the Schoenfeld residual test. | 4 |
| **Supplemental Table 3.** Correlation and Bland–Altman analyses comparing fully automated and manually edited speckle-tracking echocardiography with cardiac magnetic resonance feature tracking for strain measurements in the three cardiac chambers. | 5 |
| **Supplemental Table 4.** Reproducibility of fully-automated and manually-edited speckle tracking analysis and cardiac magnetic resonance feature tracking analysis | 6 |
| **Supplemental Table 5.** Univariable and multivariable Cox proportional analysis for determinants of major adverse cardiovascular events | 7 |
| **Supplemental Table 6.** Net reclassification improvement and integrated discrimination improvement assessing the incremental value of myocardial strain. | 8 |
| **Supplemental Figure 1.** Flow diagram for study participants. | 9 |
| **Supplemental Figure 2.** Schema of manually-edited speckle tracking analysis in the left atrium (LA). | 10 |
| **Supplemental Figure 3.** Histogram for strain distribution using fully-automated speckle tracking, manual-edited speckle tracking, and cardiac magnetic resonance feature tracking. | 11 |
| **Supplemental Figure 4.** Kaplan-Meier analyses of multi-chamber strain using fully-automated speckle tracking analysis. | 12 |
| **Supplemental Figure 5.** Kaplan-Meier analyses of multi-chamber strain using cardiac magnetic resonance feature tracking analysis. | 13 |
| **Supplemental Figure 6.** Incremental value of multi-chamber strain using an alternative sequence of addition. | 14 |
| **Supplemental Figure 7.** Decision curve analyses of the nested Cox regression models across imaging modalities. | 15 |

**Supplemental Methods**

*Definition of image quality in two-dimensional echocardiography*

In two-dimensional echocardiography, we defined the image quality based on the number of visible segments. For the left ventricle, we used the 18-segment model and categorized image quality as good (0–2 segments poorly visible), fair (3–5 segments poorly visible), poor (6-8 segments poorly visible), or extremely poor (≥9 segments poorly visible). For the left atrium and right ventricle, we divided their endocardial borders into 6 segments under the standard apical four-chamber and right ventricle-aimed apical four chamber views, respectively. Image quality was graded as either good (all segments visible), fair (1 segments poorly visible), poor (2 segments poorly visible), or extremely poor (≥3 segments poorly visible).

**Supplemental Tables**

**Supplemental Table 1.** Cutoff values of myocardial strain for patient stratification according to previous studies

| **Strain type** | **2D-STE** | **Ref** | **CMR-FT** | **Ref** |
| --- | --- | --- | --- | --- |
| LVGLS | 17.0% | [1] | 12.5% | [2] |
| LASr | 22.5% | [3] | 14.8% | [4] |
| RVfwLS | 20.0% | [5] | 17.2% | [6] |

2D-STE, two-dimensional speckle tracking echocardiography; CMR-FT, cardiovascular magnetic resonance feature tracking. See Table 1 for other abbreviations.

References:

[1] Asch FM, Miyoshi T, Addetia K, et al. Similarities and Differences in Left Ventricular Size and Function among Races and Nationalities: Results of the World Alliance Societies of Echocardiography Normal Values Study. J Am Soc Echocardiogr 2019;32:1396-1406 e1392. doi: 10.1016/j.echo.2019.08.012

[2] Wang TKM, Kwon DH, Griffin BP, Flamm SD, Popovic ZB. Defining the Reference Range for Left Ventricular Strain in Healthy Patients by Cardiac MRI Measurement Techniques: Systematic Review and Meta-Analysis. AJR Am J Roentgenol 2021;217:569-583. doi: 10.2214/AJR.20.24264

[3] Singh A, Carvalho Singulane C, Miyoshi T, et al. Normal Values of Left Atrial Size and Function and the Impact of Age: Results of the World Alliance Societies of Echocardiography Study. J Am Soc Echocardiogr 2022;35:154-164 e153. doi: 10.1016/j.echo.2021.08.008

[4] Peng J, Zhao X, Zhao L, et al. Normal Values of Myocardial Deformation Assessed by Cardiovascular Magnetic Resonance Feature Tracking in a Healthy Chinese Population: A Multicenter Study. Front Physiol 2018;9:1181. doi: 10.3389/fphys.2018.01181

[5] Addetia K, Miyoshi T, Citro R, et al. Two-Dimensional Echocardiographic Right Ventricular Size and Systolic Function Measurements Stratified by Sex, Age, and Ethnicity: Results of the World Alliance of Societies of Echocardiography Study. J Am Soc Echocardiogr 2021;34:1148-1157 e1141. doi: 10.1016/j.echo.2021.06.013

[6] Liu B, Dardeer AM, Moody WE, et al. Reference ranges and reproducibility studies for right heart myocardial deformation by feature tracking cardiovascular magnetic resonance imaging. Data Brief 2018;16:244-249. doi: 10.1016/j.dib.2017.11.037

**Supplementary Table 2**. Validation of the proportional hazard assumption using the Schoenfeld residual test.

| ***Fully automated speckle tracking echocardiography*** | | | | | | | | | | | | |
| --- | --- | --- | --- | --- | --- | --- | --- | --- | --- | --- | --- | --- |
|  | **Model 0** | | **Model 1** | | **Model 2** | | **Model3** | | **Model 4** | | **Model 5** | |
|  | chisq | P | chisq | P | chisq | P | chisq | P | chisq | P | chisq | P |
| Age | 4.69 | **0.030** | 3.87 | **0.049** | 1.54 | 0.215 | 1.50 | 0.221 | 3.86 | **0.049** | 1.50 | 0.221 |
| Sex | 5.81 | **0.016** | 4.99 | **0.025** | 5.77 | **0.016** | 5.83 | **0.016** | 5.07 | **0.024** | 5.83 | **0.016** |
| NYHA | 2.59 | 0.114 | 1.10 | 0.294 | 0.72 | 0.397 | 0.73 | 0.393 | 1.15 | 0.284 | 0.73 | 0.393 |
| CCI | 0.32 | 0.570 | 0.48 | 0.488 | 1.13 | 0.288 | 1.14 | 0.286 | 0.45 | 0.502 | 1.14 | 0.285 |
| LVGLS |  |  | 1.23 | 0.269 |  |  | 0.78 | 0.378 | 1.21 | 0.271 | 0.78 | 0.378 |
| LASr |  |  |  |  | 1.76 | 0.184 | 1.78 | 0.182 |  |  | 1.78 | 0.182 |
| RVfwLS |  |  |  |  |  |  |  |  | 0.10 | 0.755 | 0.114 | 0.735 |
| Global | 12.24 | **0.016** | 10.93 | 0.053 | 10.34 | 0.066 | 10.73 | 0.097 | 13.49 | **0.036** | 13.15 | 0.068 |
| ***Manually edited speckle tracking echocardiography*** | | | | | | | | | | | | |
|  | **Model 0** | | **Model 1** | | **Model 2** | | **Model3** | | **Model 4** | | **Model 5** | |
|  | chisq | P | chisq | P | chisq | P | chisq | P | chisq | P | chisq | P |
| Age | 3.75 | 0.053 | 2.99 | 0.084 | 0.97 | 0.325 | 1.02 | 0.313 | 2.85 | 0.091 | 1.01 | 0.315 |
| Sex | 4.73 | **0.030** | 3.92 | **0.048** | 4.17 | **0.041** | 4.13 | **0.042** | 3.98 | **0.046** | 4.12 | **0.042** |
| NYHA | 2.53 | 0.112 | 1.05 | 0.305 | 0.76 | 0.383 | 0.74 | 0.390 | 1.05 | 0.306 | 0.71 | 0.400 |
| CCI | 0.576 | 0.448 | 0.70 | 0.405 | 1.61 | 0.204 | 1.61 | 0.204 | 0.75 | 0.388 | 1.58 | 0.209 |
| LVGLS |  |  | 1.23 | 0.268 |  |  | 1.23 | 0.268 | 1.10 | 0.294 | 1.17 | 0.280 |
| LASr |  |  |  |  | 1.14 | 0.286 | 1.12 | 0.291 |  |  | 1.09 | 0.296 |
| RVfwLS |  |  |  |  |  |  |  |  | 0.11 | 0.736 | 0.08 | 0.771 |
| Global | 11.29 | **0.024** | 9.86 | 0.079 | 8.95 | 0.111 | 9.01 | 0.173 | 10.59 | 0.102 | 10.07 | 0.185 |
| ***Cardiac magnetic resonance feature tracking*** | | | | | | | | | | | | |
|  | **Model 0** | | **Model 1** | | **Model 2** | | **Model3** | | **Model 4** | | **Model 5** | |
|  | chisq | P | chisq | P | chisq | P | chisq | P | chisq | P | chisq | P |
| Age | 1.58 | 0.209 | 1.26 | 0.262 | 0.32 | 0.571 | 0.47 | 0.495 | 1.27 | 0.261 | 0.49 | 0.483 |
| Sex | 5.42 | **0.020** | 3.98 | **0.046** | 5.16 | **0.023** | 4.56 | **0.033** | 4.02 | **0.045** | 4.62 | **0.032** |
| NYHA | 2.94 | 0.086 | 1.69 | 0.194 | 1.00 | 0.318 | 1.05 | 0.306 | 1.66 | 0.183 | 1.12 | 0.290 |
| CCI | 0.957 | 0.328 | 0.91 | 0.340 | 1.95 | 0.163 | 1.68 | 0.195 | 0.83 | 0.362 | 1.61 | 0.204 |
| LVGLS |  |  | 1.40 | 0.237 |  |  | 1.36 | 0.243 | 1.16 | 0.282 | 1.29 | 0.257 |
| LASr |  |  |  |  | 2.44 | 0.118 | 1.97 | 0.160 |  |  | 1.90 | 0.169 |
| RVfwLS |  |  |  |  |  |  |  |  | 0.08 | 0.773 | 0.035 | 0.852 |
| Global | 10.09 | **0.039** | 8.64 | 0.124 | 9.71 | 0.084 | 9.24 | 0.161 | 10.12 | 0.120 | 11.82 | 0.107 |

Thejvariables incorporated in **Model 0-5** were as follows: ***Model 0***, Age +Sex +NYHA +CCI; ***Model 1***, Model 0 +LVGLS; ***Model 2***, Model 0 +LASr; ***Model 3***, Model 0 +LVGLS +LASr; ***Model 4***, Model 0 +LVGLS +RVfwLS; ***Model 5***, Model 0+ LVGLS +LASr +RVfwLS. CCI, Charlson comorbidity index; chisq: chi-squared statistic; LASr, left atrial reservoir strain; LVGLS, left ventricular global longitudinal strain; NYHA, New York Heart Association functional class; RVfwLS, right ventricular free-wall longitudinal strain.

**Supplemental Table 3.** Correlation and Bland–Altman analyses comparing fully automated and manually edited speckle-tracking echocardiography with cardiac magnetic resonance feature tracking for strain measurements in the three cardiac chambers.

|  | Pearson correlation coefficient | Mean bias | Limits of agreement | |
| --- | --- | --- | --- | --- |
|  |  |  | Upper | Lower |
| LVGLS_auto_ vs LVGLS_ft_ | 0.79 (0.76-0.82) | 2.4 (2.2-2.7) | 8.3 (7.9-8.8) | -3.5 (-4.0- -3.0) |
| LVGLS_edit_ vs LVGLS_ft_ | 0.81 (0.77-0.84) | 1.9 (1.7-2.2) | 7.6 (7.1-8.0) | -3.7 (-4.2- -3.3) |
| LASr_auto_ vs LASr_ft_ | 0.67 (0.62-0.72) | 9.7 (8.9-10.6) | 28.6 (27.1-30.0) | -9.2 (-10.6- -7.7) |
| LASr_edit_ vs LASr_ft_ | 0.67 (0.62-0.71) | 6.7 (6.0-7.3) | 22.0 (20.8-23.1) | -8.7 (-9.8- -7.5) |
| RVfwLS_auto_ vs RVfwLS_ft_ | 0.48 (0.41-0.55) | -0.1 (-0.8-0.6) | 14.4 (13.2-15.5) | -14.6 (-15.7- -13.3) |
| RVfwLS_edit_ vs RVfwLS_ft_ | 0.52 (0.45-0.58) | -0.6 (-1.2-0.0) | 13.1 (12.0-14.1) | -14.3 (-15.4- -13.2) |

Data are reported as estimate (95% confidence intervals). auto, fully automated; edit, manually edited; ft, feature tracking; LVGLS, left ventricular global longitudinal strain; LASr, left atrial reservoir strain; RVfwLS, right ventricular free-wall longitudinal strain.

**Supplemental Table 4.** Reproducibility of fully automated and manually edited speckle tracking and cardiac magnetic resonance feature tracking analyses.

| **Intra-rater ICC** | **LVGLS** | **LASr** | **RVfwLS** |
| --- | --- | --- | --- |
| Fully-automated speckle tracking | 1.0 | 1.0 | 1.0 |
| Manually-edited speckle tracking | 0.99 | 0.95 | 0.88 |
| Cardiac magnetic resonance feature tracking | 0.92 | 0.89 | 0.81 |
|  |  |  |  |
| **Inter-rater ICC** | **LVGLS** | **LASr** | **RVfwLS** |
| Fully-automated speckle tracking | 1.0 | 1.0 | 1.0 |
| Manually-edited speckle tracking | 0.97 | 0.83 | 0.85 |
| Cardiac magnetic resonance feature tracking | 0.92 | 0.90 | 0.80 |

ICC, intraclass correlation coefficients; LVGLS, left ventricular global longitudinal strain; LASr, left atrial reservoir strain; RVfwLS, right ventricular free-wall longitudinal strain.

**Supplemental Table 5.** Univariable and multivariable Cox analysis for determinants of major adverse cardiovascular events.

| Varables | HR | 95% CI | p-value |
| --- | --- | --- | --- |
| Age | 1.04 | 1.02 – 1.06 | <0.001 |
| Male | 0.85 | 0.54 – 1.34 | 0.5 |
| Body surface area | 0.31 | 0.10 – 0.97 | 0.044 |
| NYHA III or IV | 4.06 | 2.44 – 6.77 | <0.001 |
| CCI | 1.18 | 1.09 – 1.28 | <0.001 |
| Heart rate | 1.02 | 1.00 – 1.03 | 0.052 |
| Systolic blood pressure | 0.98 | 0.97 – 0.99 | <0.001 |
| Diastolic blood pressure | 0.96 | 0.94 – 0.98 | <0.001 |
| LVGLS_auto_ | 0.87 | 0.83 – 0.91 | <0.001 |
| LASr_auto_ | 0.91 | 0.88 – 0.93 | <0.001 |
| RVfwLS_auto_ | 0.94 | 0.90 – 0.97 | <0.001 |
| LVGLS_edit_ | 0.86 | 0.82 – 0.91 | <0.001 |
| LASr_edit_ | 0.89 | 0.86 – 0.92 | <0.001 |
| RVfwLS_edit_ | 0.92 | 0.88 – 0.96 | <0.001 |
| LVGLS_ft_ | 0.86 | 0.81 – 0.91 | <0.001 |
| LASr_ft_ | 0.88 | 0.85 – 0.92 | <0.001 |
| RVfwLS_ft_ | 0.93 | 0.90 – 0.96 | <0.001 |
| **Adjustments for age, sex, NYHA, CCI** | | | |
| LVGLS_auto_ | 0.88 | 0.83 – 0.93 | <0.001 |
| LASr_auto_ | 0.91 | 0.89 – 0.94 | <0.001 |
| RVfwLS_auto_ | 0.94 | 0.90 – 0.97 | 0.001 |
| LVGLS_edit_ | 0.87 | 0.82 – 0.92 | <0.001 |
| LASr_edit_ | 0.90 | 0.87 – 0.93 | <0.001 |
| RVfwLS_edit_ | 0.91 | 0.87 – 0.95 | <0.001 |
| LVGLS_ft_ | 0.87 | 0.82 – 0.92 | <0.001 |
| LASr_ft_ | 0.88 | 0.84 – 0.92 | <0.001 |
| RVfwLS_ft_ | 0.93 | 0.90 – 0.96 | <0.001 |

HR, Hazard Ratio, CI, Confidence Interval. See other abbreviations in Table 1.

**Supplemental Table 6.** Net reclassification improvement and integrated discrimination improvement assessing the incremental value of myocardial strain.

| ***Fully automated speckle tracking echocardiography*** | | |
| --- | --- | --- |
| **Model 0** Age + Sex^*^ + NYHA +CCI | IDI | NRI |
| **Model 1** Model 0 +***LVGLS*** | **0.045** (0.010-0.099) | **0.276** (0.074-0.412) |
| **Model 2** Model 0 +***LASr*** | **0.092** (0.040-0.160) | **0.336** (0.187-0.491) |
| **Model 3-1** Model 1 + ***LASr*** | **0.047** (0.011-0.099) | **0.215** (0.021-0.379) |
| **Model 3-2** Model 2 + ***LVGLS*** | 0.000 (-0.003-0.017) | 0.116 (-0.224-0.256) |
| **Model 4.** Model 1 +***RVfwLS*** | 0.005 (-0.005-0.037) | 0.078 (-0.170-0.287) |
| **Model 5-1** Model 3-1+ ***RVfwLS*** | 0.000 (-0.010-0.037) | -0.049 (-0.162-0.207) |
| **Model 5-2** Model 4 + ***LASr*** | **0.042** (0.006-0.095) | **0.233** (0.020-0.407) |
| ***Manually edited speckle tracking echocardiography*** | | |
| **Model 1** Model 0 +***LVGLS*** | **0.050** (0.017-0.104) | **0.297** (0.109-0.419) |
| **Model 2** Model 0 +***LASr*** | **0.099** (0.049-0.171) | **0.362** (0.154-0.517) |
| **Model 3-1** Model 1 + ***LASr*** | **0.048** (0.009-0.121) | **0.257** (0.075-0.431) |
| **Model 3-2** Model 2 + ***LVGLS*** | -0.001 (-0.007-0.024) | -0.050 (-0.179-0.233) |
| **Model 4.** Model 1 +***RVfwLS*** | 0.012 (-0.002-0.054) | 0.109 (-0.111-0.246) |
| **Model 5-1** Model 3-1+ ***RVfwLS*** | 0.005 (-0.004-0.039) | 0.055 (-0.204-0.213) |
| **Model 5-2** Model 4 + ***LASr*** | **0.041** (0.004-0.109) | **0.219** (0.035-0.397) |
| ***Cardiac magnetic resonance feature tracking*** | | |
| **Model 1** Model 0 +***LVGLS*** | **0.048** (0.006-0.102) | **0.294** (0.082-0.420) |
| **Model 2** Model 0 +***LASr*** | **0.080** (0.034-0.140) | **0.342** (0.180-0.475) |
| **Model 3-1** Model 1 + ***LASr*** | **0.032** (0.001-0.082) | **0.267** (0.109-0.419) |
| **Model 3-2** Model 2 + ***LVGLS*** | 0.000 (-0.009-0.032) | 0.029 (-0.200-0.207) |
| **Model 4.** Model 1 +***RVfwLS*** | 0.008 (-0.004-0.038) | 0.063 (-0.168-0.236) |
| **Model 5-1** Model 3-1+ ***RVfwLS*** | 0.001 (-0.003-0.022) | 0.046 (-0.210-0.204) |
| **Model 5-2** Model 4 + ***LASr*** | 0.025 (-0.002-0.069) | **0.270** (0.043-0.429) |

*All models were constructed with stratification by sex. CCI, Charlson comorbidity index; IDI, integrated discrimination improvement; LASr, left atrial reservoir strain; LVGLS, left ventricular global longitudinal strain; NRI, net reclassification improvement; NYHA, New York Heart Association functional class; RVfwLS, right ventricular free-wall longitudinal strain.

**Supplemental Figures**

**Supplemental Figure 1.** Flow diagram for study participants.

**
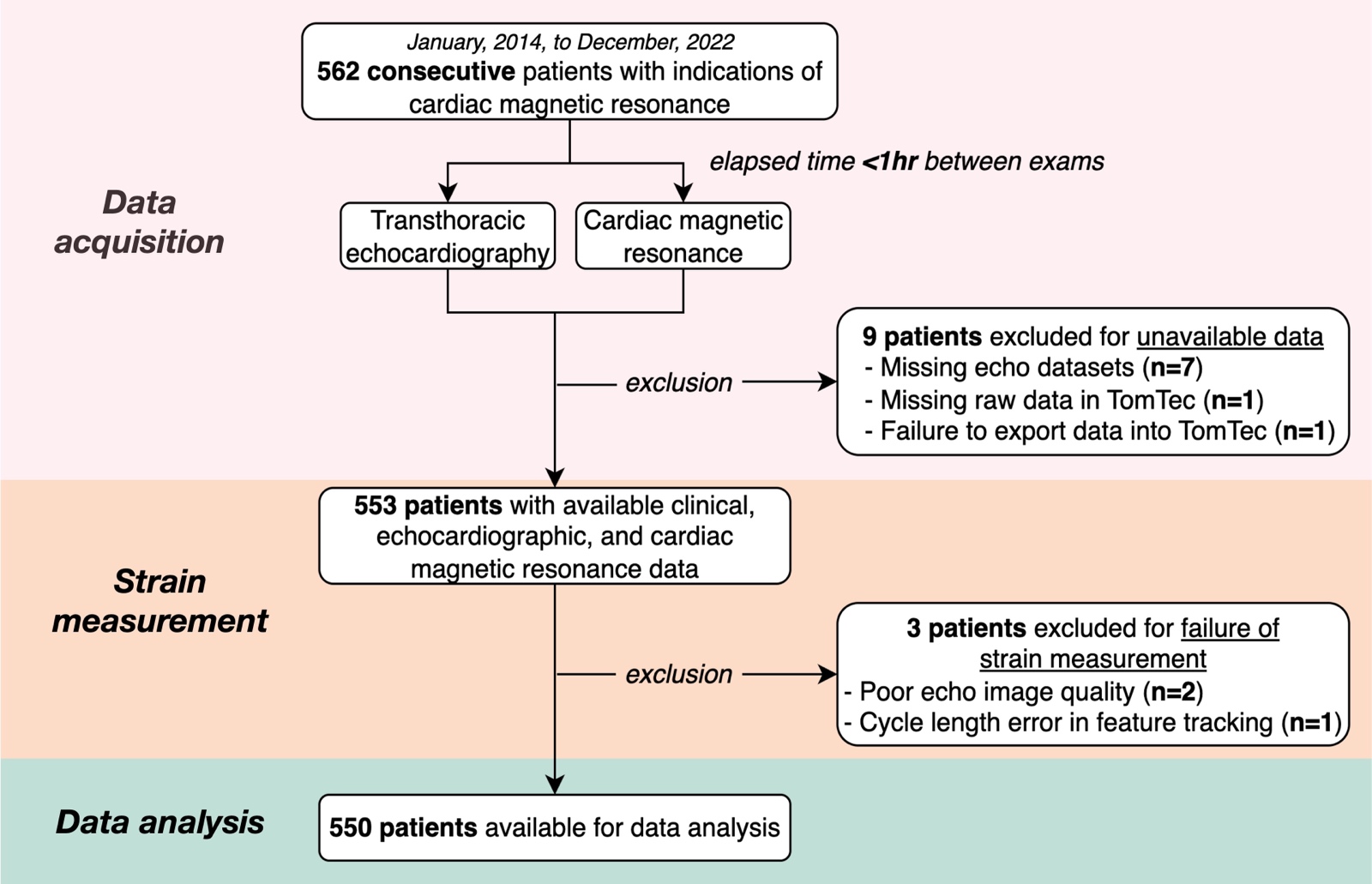
**

**Supplemental Figure 2.** Schema of manually-edited speckle tracking analysis in the left atrium. (A) The software automatically detected the endocardial border and region of interest, providing speckle-tracking strain values. (B) Manual correction of the second cardiac cycle according to the left ventricular end-diastolic phase. (C) Manual correction of the endocardial border. The software then performed a second speckle tracking analysis and generated a new strain value.


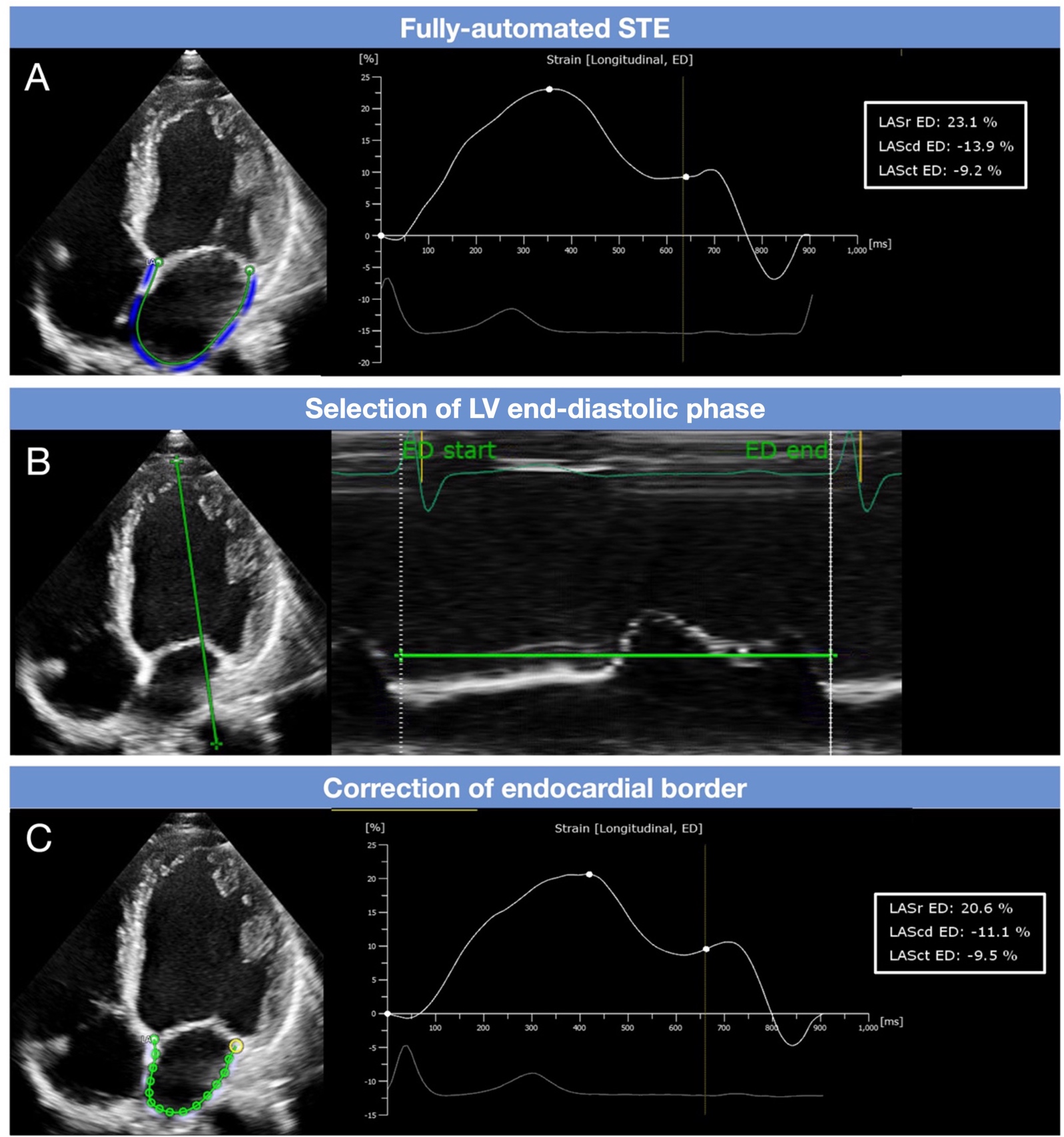


**Supplemental Figure 3.** Histograms for strain distribution using fully-automated speckle tracking echocardiography (A), manually-edited speckle tracking echocardiography (B), and cardiac magnetic resonance feature tracking (C). LVGLS, left ventricular global longitudinal strain; LASr, left atrial reservoir strain; RVfwLS, right ventricular free-wall longitudinal strain.


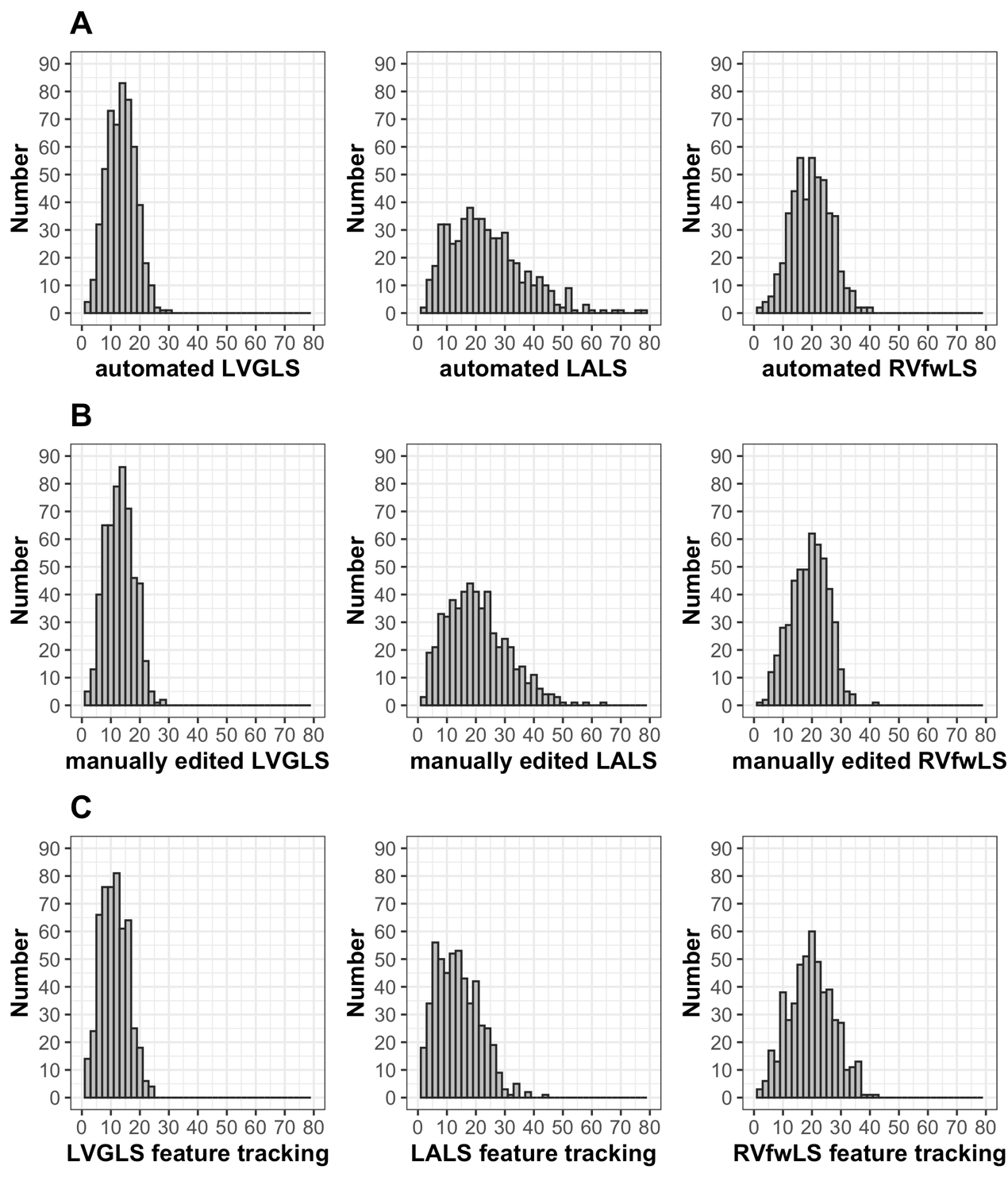


**automated LASr**

**manually edited LASr**

**LASr feature tracking**


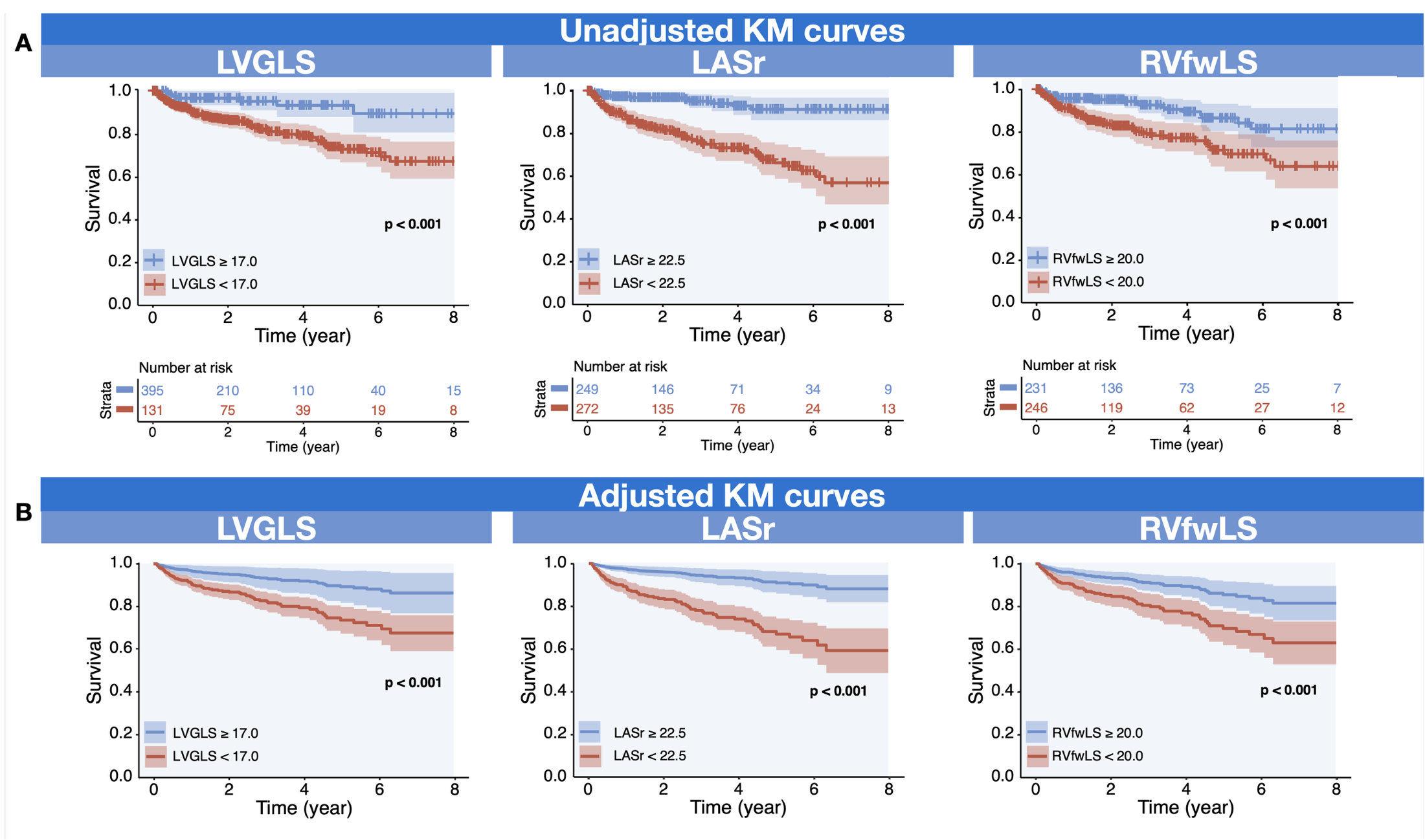
**Supplemental Figure 4.** **Kaplan-Meier analyses of multi-chamber strain using fully-automated speckle tracking analysis.**
Patients with lower left ventricular global longitudinal strain (LVGLS), left atrial reservoir strain (LASr), and right ventricular free-wall longitudinal strain (RVfwLS) were associated with higher rates of major adverse cardiovascular events before (A) and after adjustment (B). Adjustments were made for age, sex, New York Heart Association functional class, and Charlson’s comorbidity index. KM, Kaplan-Meier.


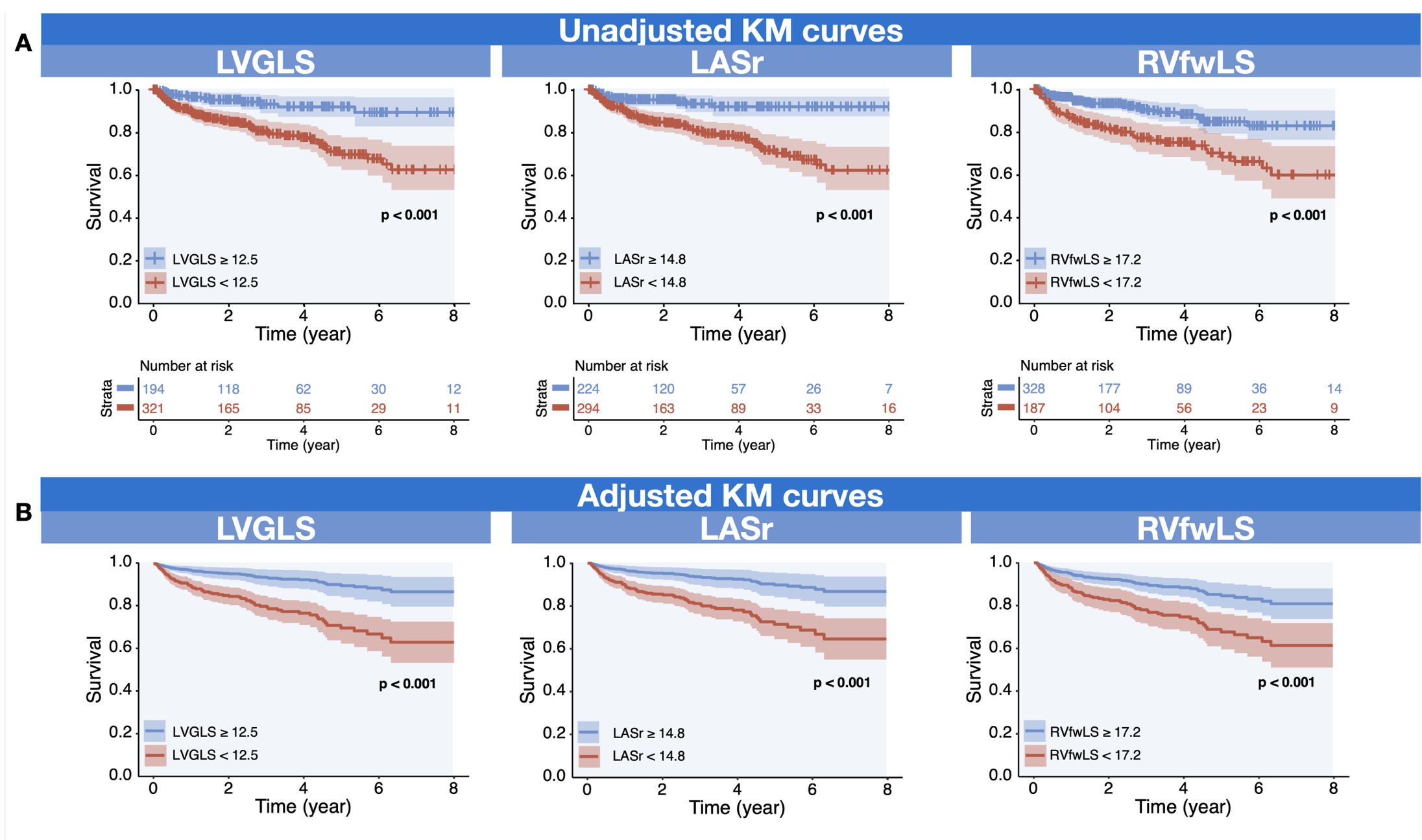
**Supplemental Figure 5. Kaplan-Meier analyses of multi-chamber strain using cardiac magnetic resonance feature tracking analysis**.

Patients with lower left ventricular global longitudinal strain (LVGLS), left atrial reservoir strain (LASr), and right ventricular free-wall longitudinal strain (RVfwLS) were associated with higher rates of major adverse cardiovascular events before (A) and after adjustment (B). Adjustments were made for age, sex, New York Heart Association functional class, and Charlson’s comorbidity index. KM, Kaplan-Meier.

**Supplemental Figure 6. Incremental value of multi-chamber strain using an alternative sequence of addition.**
Model 0 included age, New York Heart Association (NYHA) functional class, Charlson’s comorbidity index (CCI), and stratification by sex. Across all imaging modalities, left atrial reservoir strain (LASr) provided incremental value when added to Model 0. However, neither left ventricular global longitudinal strain (LVGLS) nor right ventricular free-wall longitudinal strain (RVfwLS) showed additional value beyond Model 0 +LASr for major adverse cardiovascular events. CMR-FT, cardiac magnetic resonance feature tracking; 2D-STE, two-dimensional speckle tracking echocardiography.


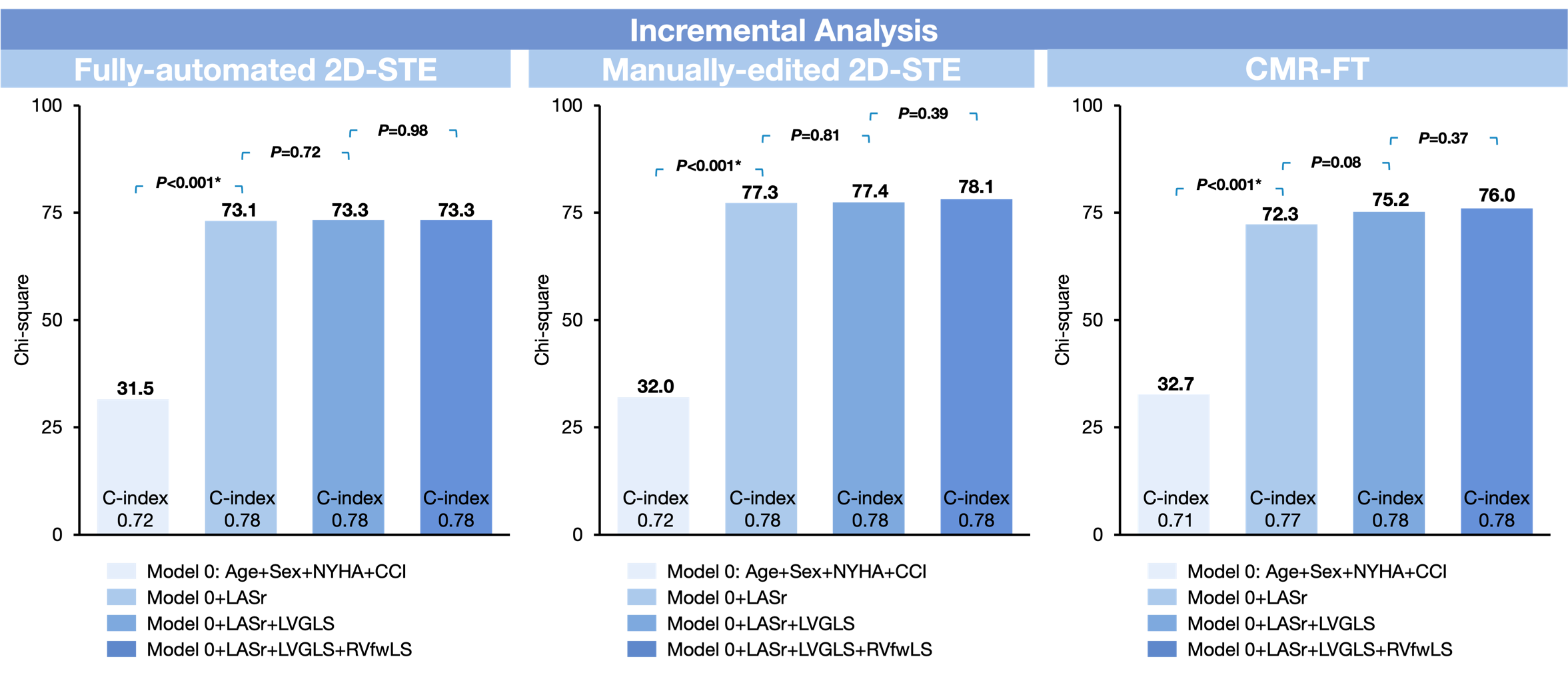


**Supplemental Figure 7.** Decision curve analyses of the nested Cox regression models across imaging modalities. All models were constructed with stratification by sex. CCI, Charlson’s comorbidity index; CMR-FT, cardiac magnetic resonance feature tracking; LASr, LA reservoir strain; LVGLS, LV global longitudinal strain; NYHA, New York Heart Association functional class; RVfwLS, RV free-wall longitudinal strain; 2D-STE, two-dimensional speckle tracking echocardiography.

**
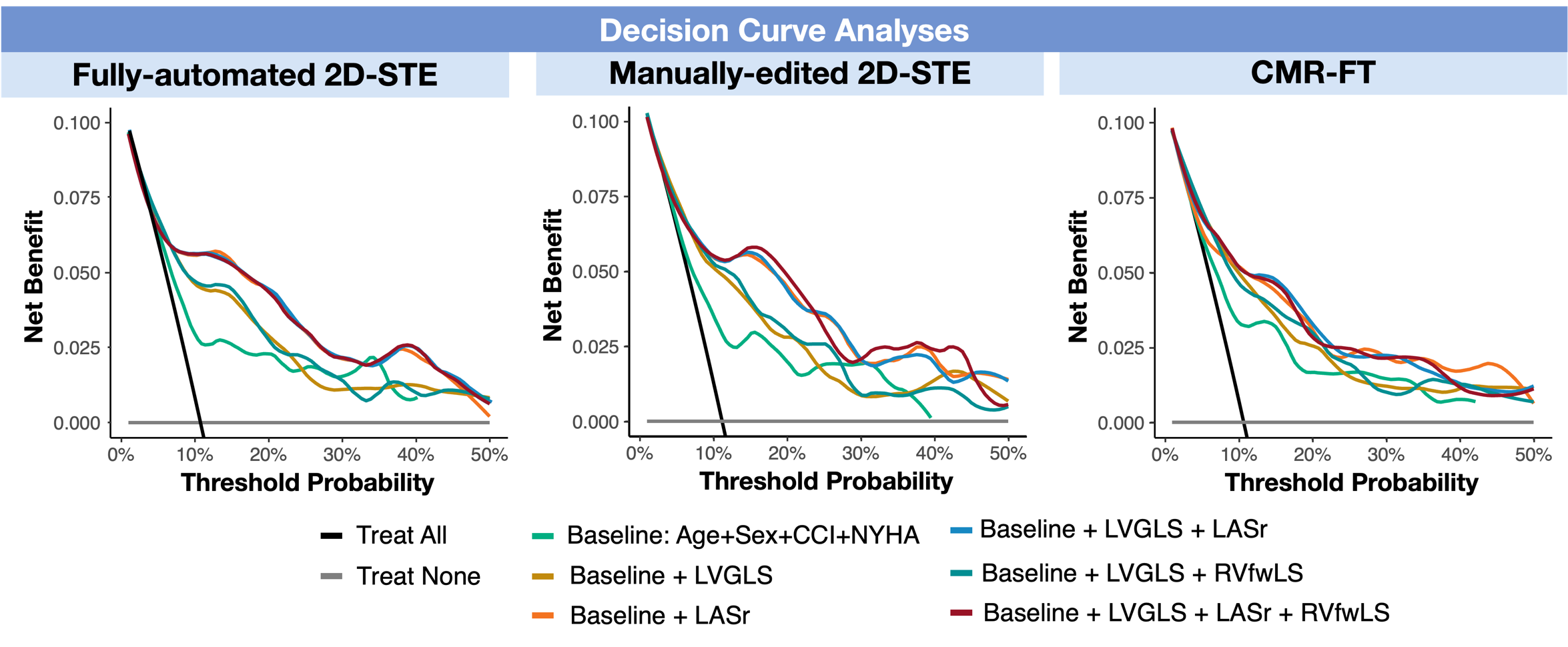
**
